# Decreased embryo developmental potential and lower cumulative pregnancy rate in men with multiple morphological abnormalities of the sperm flagella

**DOI:** 10.3389/fendo.2024.1377780

**Published:** 2024-04-30

**Authors:** Rui Long, Meng Wang, Juepu Zhou, Ruolin Mao, Cheng Wang, Longjie Gu, Yinwei Chen, Lei Jin, Lixia Zhu

**Affiliations:** Reproductive Medicine Center, Tongji Hospital, Tongji Medical College, Huazhong University of Science and Technology, Wuhan, China

**Keywords:** MMAF, ICSI, WES, mutations, male infertility

## Abstract

**Objective:**

Multiple morphological abnormalities of the sperm flagella (MMAF) is characterized by abnormal flagellar phenotypes, which is a particular kind of asthenoteratozoospermia. Previous studies have reported a comparable intracytoplasmic sperm injection (ICSI) outcome in terms of fertilization rate and clinical pregnancy rate in patients with MMAF compared with those with no MMAF; however, others have conflicting opinions. Assisted reproductive technology (ART) outcomes in individuals with MMAF are still controversial and open to debate.

**Methods:**

A total of 38 patients with MMAF treated at an academic reproductive center between January 2014 and July 2022 were evaluated in the current retrospective cohort study and followed up until January 2023. Propensity score matching was used to adjust for the baseline clinical characteristics of the patients and to create a comparable control group. The genetic pathogenesis of MMAF was confirmed by whole exome sequencing. The main outcomes were the embryo developmental potential, the cumulative pregnancy rate (CLPR), and the cumulative live birth rate (CLBR).

**Results:**

Pathogenic variants in known genes of *DNAH1*, *DNAH11*, *CFAP43*, *FSIP2*, and *SPEF2* were identified in patients with MMAF. Laboratory outcomes, including the fertilization rate, 2PN cleavage rate, blastocyst formation rate, and available blastocyst rate, followed a trend of decline in the MMAF group (*p* < 0.05). Moreover, according to the embryo transfer times and complete cycles, the CLPR in the cohort of MMAF was lower compared with the oligoasthenospermia pool (*p* = 0.033 and *p* = 0.020, respectively), while no statistical differences were observed in the neonatal outcomes.

**Conclusion:**

The current study presented decreased embryo developmental potential and compromised clinical outcomes in the MMAF cohort. These findings may provide clinicians with evidence to support genetic counseling and clinical guidance in specific patients with MMAF.

## Introduction

Asthenoteratozoospermia refers to the decrease or lack of motile sperm and combines with abnormal sperm morphology, which becomes one of the most common factors leading to male infertility (1, 2). Multiple morphological abnormalities of the sperm flagella (MMAF), a specific kind of asthenoteratozoospermia, is featured by aberrant flagellar phenotypes with absent, short, bent, coiled, and/or irregular flagella, which can be easily identified by light microscopy (3). Mammalian sperm flagella consist of a central structure called the axoneme and peri-axonemal structures (the mitochondrial sheath, the fibrous sheath, and the outer dense fibers) (4). The axoneme comprises a central pair of microtubules surrounded by nine peripheral doublet microtubules, which terms a (9 +2) structure (5). Severe ultrastructural abnormalities are observed by electronic microscopy in the flagellar of patients with MMAF, such as a lack of the central pair, with missing or disorganized peripheral doublet microtubules and missing dynein arms (6), which results in male infertility due to impaired or absent sperm motility.

Since first defined in 2014 (3), the genetic investigation of MMAF has been continuously explored. To date, nearly 40 genes have been identified that account for 30%–60% of individuals with MMAF (6). The mutations in *DNAH1*, *CFAP43*, and *CFAP44* were reported as recurrent causes of MMAF. *DNAH1* encodes axonemal inner arm dynein, which is indispensable for flagellar beating (6). *CFAP43* and *CFAP44* relate to the cilia- and flagella-associated proteins (CFAPs) with WD repeat domains (WDRs) that locate in the inner dynein arm complex tether/tether head, involved in protein interactions (7). Male *Cfap43^−/−^* and *Cfap44^−/−^* mice are sterile and present peri-axonemal and axonemal defects in sperm flagella (7). Furthermore, *DNAH2*, *DNAH6*, *DNAH17*, *CFAP65*, *CFAP70*, *CEP135*, *TTC29*, and *SPEF2*, which mainly encode for sperm components, were also mentioned in a minority of patients with MMAF (6).

Considering that flagellar defects appear to influence intracytoplasmic sperm injection (ICSI) outcomes and fetal development (8, 9), studies focusing on the ICSI outcomes and prognosis for patients with MMAF have been reported. Several studies indicated a positive ICSI outcome in the MMAF cohort (4, 10, 11), and others have opposite opinions (9, 12). However, the sample size of these studies was too small to draw convincing conclusions. Furthermore, the neonatal outcomes and the information regarding the mutations of patients with MMAF were incomplete. Therefore, it is necessary to present a general overview of the comprehensive ART outcomes of patients with MMAF and thus provide accurate guidance for clinicians. In this context, causative genetic variants in individuals with MMAF were identified by whole exome sequencing (WES) to confirm the genetic pathogenesis of MMAF. Furthermore, an extensive evaluation of ART outcomes, including *in vitro* fertilization (IVF)/ICSI outcomes, IVF/ICSI success chance, donor semen attempt, neonatal outcomes, and time costs on achieving pregnancy, was conducted in this enlarged MMAF cohort. To create a highly comparable control group, the propensity score matching (PSM) method, which can minimize the discrepancies between the different groups of patients, was performed. The propensity score aims to remove the effects of confounding in multiple clinical and genetic analyses. The current study is designed to provide precise genetic counseling and offer individual ART instruction for patients with MMAF.

## Materials and methods

### Study participants and study design

**Figure 1:**
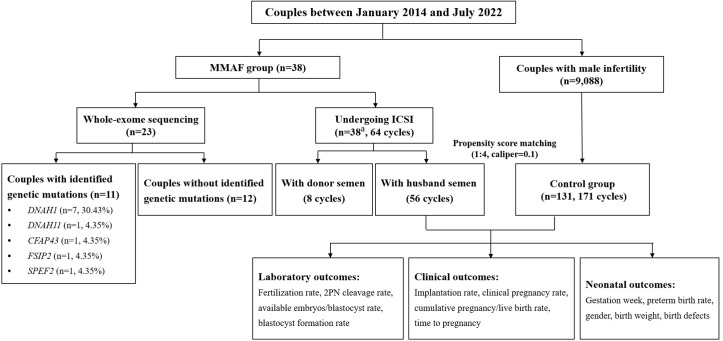
Flowchart of the study. ICSI, intracytoplasmic sperm injection; MMAF, multiple morphological abnormalities of the sperm flagella; 2PN, 2 pronuclei embryos. ^a^Including two patients who only used donor semen throughout the whole ICSI procedure.

A cohort of 38 Chinese infertile men with MMAF were enrolled from January 2014 to July 2022 at the Reproductive Medicine Center, Tongji Hospital, Tongji Medical College, Huazhong University of Science and Technology in Wuhan, China. Individuals with MMAF were recruited based on a typical MMAF phenotype (abnormal sperm flagella, including short, absent, coiled, bent, and/or irregular flagella). Couples with male infertility caused by oligoasthenozoospermia undergoing ICSI treatments over the same period were included as the control pool. Oligoasthenozoospermia is characterized by a sperm count of less than 15 million per milliliter and a proportion of progressively motile spermatozoa that is less than 32%. Meanwhile, abnormal chromosome karyotype, azoospermia, total fertilization failure (TFF), donor semen cycles, and pre-implantation genetic testing (PGT) cycles were excluded from the control pool. PSM based on basic clinical characteristics was performed by a logistic regression model (matching ratio = 1:4, caliper = 0.1) from the oligoasthenospermia pool. The ART outcomes, including laboratory outcomes, clinical outcomes, and neonatal outcomes, were compared between individuals with MMAF and the oligoasthenospermia group. The detailed flowchart is presented in **Figure 1**.

This study was approved by the Ethical Committee of Tongji Hospital (TJ-IRB20211280). Informed consent was obtained from all the participants.

### Sperm analysis and morphological assessment

All the semen samples were collected by masturbation after 2–7 days of sexual abstinence. Semen samples were examined after liquefaction for 30 min at 37°C. Manual Papanicolaou sperm staining and computer-assisted sperm analysis (CASA) system (BEION S3-3, V4.20, BEION, Shanghai, China) were used in semen analysis. The assessment of semen parameters, including volume, concentration, motility, and morphology, was based on the WHO criteria (5th edition) (13). Morphological alterations observed under an optical microscope, including abnormal sperm flagella, such as short, absent, coiled, bent, and/or irregular flagella, were recognized as MMAF. Sperm analysis and morphological assessment were performed twice to obtain accurate diagnoses for patients with MMAF.

### WES and bioinformatics analysis

WES and bioinformatics analysis were performed according to our previously described protocols (14). Briefly, genomic DNA was extracted from peripheral blood samples of 23 patients with MMAF, and the exome was enriched using the Agilent SureSelect Human All Exon Kit, and next-generation sequencing was conducted on the Illumina HiSeq X-TEN platform. Then, the obtained raw data were mapped to the human genome reference sequence (hg19/GRCh37) by the Burrows-Wheeler Aligner software. The DNA sequence variants were detected by the Genome Analysis Toolkit (GATK) software and functionally annotated by ANNOVAR software with a variety of bioinformatic databases, including 1000 Genomes (http://www.1000genomes.org/data), gnomAD (http://gnomad-sg.org/), Sorting Intolerant From Tolerant (SIFT, http://sift.bii.astar.edu.sg/), Polymorphism Phenotyping (Polyphen-2, http://genetics.bwh.harvard.edu/pph2/), Mutation Taster (http://www.mutaiontaster.org/), and Mutation Assessor (http://mutationassessor.org/). Candidate pathogenic mutations identified in the participants were validated by Sanger sequencing conducted on ABI PRISM 3500 Genetic Analyzer (Applied Biosystems, Foster City, CA).

### Scanning and transmission electron microscopy

The sperm samples were fixed with 2.5% glutaraldehyde at 4°C for at least 2 h and washed three times with 0.1 M phosphate buffer (PB, pH 7.4). After being post-fixed in 1% osmic acid for 1 h at 4°C, gradient dehydration was performed with graded ethanol and isoamyl acetate. For scanning electron microscopy (SEM), all of the samples were mounted on aluminum stubs, sputter-coated by an ionic sprayer meter, and observed by SEM (SU8100, HITACHI, Japan) at 10 kV. For transmission electron microscopy (TEM), the specimens were infiltrated with 1:1 acetone and SPI-CHEM resin for 4 h at 37°C, embedded in EMBed 812 (SPI, 90529-77-4) overnight at 37°C, and then polymerized for 48 h at 60°C. The prepared samples were cut to 70-nm ultrathin sections and collected onto 150-mesh cuprum grids followed by counterstaining with 2% uranium acetate and 2.6% lead citrate. The specimens were observed and photographed by TEM (HT7800, HITACHI, Japan) at 80 kV.

### Ovarian stimulation protocol, oocyte retrieval, ICSI procedure, and embryo transfer

Patients were submitted to standard controlled ovarian hyperstimulation (COH) protocols depending on the age, the ovarian reserve, and previous ovarian response to gonadotrophins, which were described in our previous studies (15, 16). Subsequently, an ultrasound was performed to record the follicular response. Recombinant human chorionic gonadotropin (hCG) was intramuscularly administered when at least two follicles were ≥18 mm in diameter. Oocyte retrieval was performed 36–38 h later under ultrasound guidance. During the ICSI operation, for patients with enough motile sperm, normal morphological sperm were selected for injection. If embryologists could not find enough motile spermatozoa, the sperm activator named SpermMobil (Gynemed, Germany) was applied to increase the motility of sperm and the number of motile spermatozoa for injection. If no motile sperm was detected after using SpermMobil, oocyte cryopreservation or immotile sperm injection was conducted with patient consent. After fertilization, the process of embryo culture was managed according to our previously reported protocol (17). The best embryos were selected for transfer on day 3 after oocyte retrieval. Supernumerary good-quality embryos were frozen on day 3 or further cultured to day 5 or 6 for cryopreservation. Frozen–thawed embryo transfer was conducted after priming the uterus with estrogen.

### Outcome assessment

The ART outcomes of the current study mainly covered laboratory outcomes, clinical outcomes, and neonatal outcomes. The fertilization rate was defined as the number of 2 pronuclei (2PN) zygotes divided by the number of the matured oocytes; the 2PN cleavage rate was defined as the number of 2PN cleaved embryos divided by the number of 2PN zygotes; the available embryo rate referred to the ratio of the number of available embryos (including transfer, freezing, and further culture) to the number of 2PN cleaved embryos and the late-cleaved embryos; the blastocyst formation rate was the number of blastocysts on day 5 or 6 divided by the number of day 3 embryos for further culture; the available blastocyte rate was defined as the number of frozen blastocytes divided by the number of day 3 embryos for extended culture. Implantation rate referred to the ratio of the number of gestational sacs to the number of embryos transferred. Biochemical pregnancy was confirmed with an elevated serum hCG level on day 14 after embryo transfer, and clinical pregnancy was defined as the presence of an intrauterine gestational sac with an active fetal heart by transvaginal ultrasound after 4–6 weeks of embryo transfer. The ART and neonatal outcomes were followed up until January 2023.

The cumulative pregnancy rate (CLPR) and the cumulative live birth rate (CLBR) were calculated as the cumulative number of pregnancy/live births up to the specific complete treatment cycle/embryo transfer time, divided by the number of women who started their first ART cycle during the study period. The complete cycle contains the fresh and all frozen embryo transfers following one ovarian stimulation. Log-rank test and Kaplan–Meier curves with pregnancy and live birth were applied to illustrate differences among groups (18).

### Statistical analysis

SPSS (version 22.0) was utilized to perform the current statistical analyses. All continuous data presenting nonnormal distribution were confirmed via Mann–Whitney *U* test and displayed as the median [interquartile range (IQR)]; otherwise, Student’s *t*-test was performed for normally distributed data. Moreover, the categorical data are shown as the number of cases and frequency (percentage), with Fisher’s exact test or Pearson’s *χ* ^2^ test. Statistical significance was defined as a two-tailed *p*-value <0.05.

PSM was performed to create a highly comparable control pool by a logistic regression model via SPSS software (version 22.0) (matching ratio = 1:4, caliper = 0.1). Male age, female age, female basal follicle-stimulating hormone (FSH) levels, female body mass index (BMI), antral follicle count (AFC), female anti-Müllerian hormone (AMH) levels, infertility type, infertility duration, estradiol level on the hCG day, progesterone level on the hCG day, endometrial thickness on the hCG day, and the number of large follicles were included in the model to balance the baseline characteristics and sample sizes between the MMAF cohort and the oligoasthenospermia pool.

## Results

### Baseline characteristics

A cohort of 38 patients with MMAF phenotype, between January 2014 and July 2022 at the Reproductive Medicine Center, Tongji Hospital, Tongji Medical College, Huazhong University of Science and Technology, were enrolled in the MMAF group. According to PSM, 131 couples were included in the oligoasthenospermia group at a ratio of 1:4. All of the patients had a normal chromosome karyotype. No significant differences were found between the matched cohorts in terms of characteristics (**Supplementary Table 1**).

### Ultrastructural anomalies of MMAF sperm

**Figure 2:**
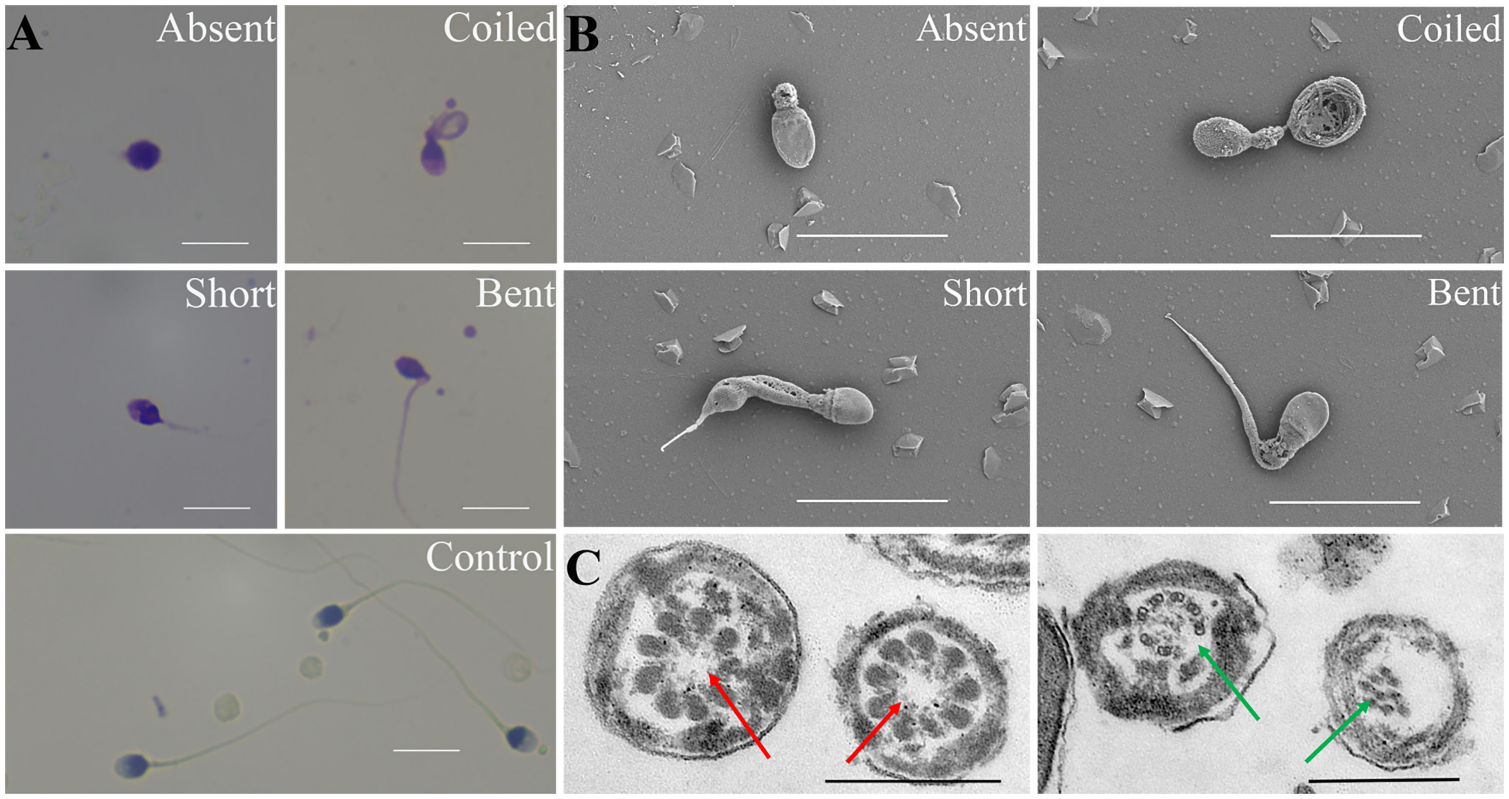
Morphological assessment of the MMAF phenotype. **(A)** Papanicolaou staining results show multiple morphological abnormalities in patients with MMAF, including absent, short, coiled, and bent tails, compared with healthy donors. **(B)** Scanning electron microscope images of sperms of individuals with MMAF. **(C)** Transmission electron micrograph of the flagellar transversal section in individuals with MMAF. Red arrows show the absent central complex and that the axonemal structure was disorganized (green arrows). White scale bars: 10 μm. Black scale bars: 500 nm. MMAF, multiple morphological abnormalities of the sperm flagella.

Typical MMAF phenotype (abnormal sperm flagella, including short, absent, coiled, bent, and/or irregular flagella) were observed by light microscopy and SEM (**Figures 2A, B**). TEM was performed to explore the ultrastructural aspects of sperm in patients with MMAF. Central complex defects (9 + 0 structure) and a disorganized axonemal structure were found in individuals with MMAF (**Figure 2C**).

### Genetic mutations in the MMAF cohort

Among the 38 included men with MMAF, 23 were subjected to WES, of whom 11 (47.8%) were verified as having bi-allelic identified variants in five genes known to be associated with the MMAF phenotype (**Supplementary Table 2**). A total of seven patients had *DNAH1* heterozygous/homozygous mutations, three patients had heterozygous mutations in either *DNAH11*, *CFAP43*, or *FSIP2*, and one patient was identified with a homozygous mutation in *SPEF2*. All the candidate pathogenic mutations identified in the participants were validated by Sanger sequencing.

### General ART outcomes

The 38 couples underwent a total of 64 ovarian stimulation cycles, including 56 cycles with the husband’s semen and 8 cycles with a donor semen. **Table 1** presents the laboratory outcomes in the MMAF couples with the husband’s semen versus the oligoasthenospermia couples. The mature oocyte rate and available embryo rate were similar between the patients with MMAF and those with oligoasthenospermia. However, the fertilization rate, 2PN cleavage rate, blastocyst formation rate, and available blastocyte rate in the MMAF group were all significantly lower than those in the oligoasthenospermia group (58.3% *vs.* 71.3%, *p* < 0.001; 94.8% *vs.* 97.1%, *p* = 0.030; 50.9% *vs.* 68.7%, *p* < 0.001; 33.8% *vs.* 45.1%, *p* = 0.001, respectively).

**Table 1:** Laboratory outcomes of MMAF and the oligoasthenospermia groups.

	MMAF ^a^	Oligoasthenospermia	*p*-value
**No. of patients**	36	131	–
**No. of ART cycles**	56	171	–
**No. of oocytes retrieved**	772	2160	–
**Mature oocyte rate**	81.3 (628/772)	81.8 (1,766/2,160)	0.800
**Fertilization rate**	58.3 (366/628)	71.3 (1,259/1,766)	**<0.001**
**2PN cleavage rate**	94.8 (347/366)	97.1 (1,223/1,259)	**0.030**
**Available embryo rate**	93.3 (332/356)	90.3 (1,126/1,247)	0.086
**Blastocyst formation rate**	50.9 (137/269)	68.7 (644/937)	**<0.001**
**Available blastocyst rate**	33.8 (91/269)	45.1 (423/937)	**0.001**

Categorical variables are presented as % (n/N). Fisher’s exact test or Pearson’s χ^2^ test was used to compare the differences between the two groups.

a Only included IVF/ICSI with the husband’s semen.

MMAF, multiple morphological abnormalities of the sperm flagella; ART, assisted reproductive technology; 2PN, 2 pronuclei embryos; IVF, *in vitro* fertilization; ICSI, intracytoplasmic sperm injection.

Bold values were used to emphasize the statistical significance (P<0.05).

The data on clinical outcomes are shown in **Table 2**. A total of 76 and 280 embryos were transferred in the MMAF and the oligoasthenospermia groups, respectively. Although no significant differences were observed in the implantation rate, biochemical pregnancy rate, clinical pregnancy rate, early miscarriage rate, and live birth rate between these two groups (*p* > 0.05), an apparently decreased trend was shown in the cohort of MMAF. Moreover, the average number of complete cycles for patients in achieving successful pregnancy was obviously higher in the MMAF group (1.58 *vs*. 1.17, *p* = 0.034).

**Table 2:** Clinical outcomes of MMAF and the oligoasthenospermia groups.

	MMAF ^a^	Oligoasthenospermia	*p*-value
**No. of patients**	36	131	–
**No. of ET cycle**	62	223	–
**No. of embryos transferred**	76	280	–
**Implantation rate**	36.8 (28/76)	43.2 (121/280)	0.318
**Biochemical pregnancy rate**	54.8 (34/62)	57.4 (128/223)	0.719
**Clinical pregnancy rate**	41.9 (26/62)	52.9 (118/223)	0.126
**Early miscarriage rate**	11.5 (3/26)	18.6 (22/118)	0.562
**Live birth rate**	24.2 (15/62)	34.5 (77/223)	0.124
**Average no. of complete cycles per patient**	1.56	1.31	0.101
**Average no. of complete cycles in patients achieving pregnancy**	1.58	1.17	**0.034**

Categorical variables are presented as % (n/N). Fisher’s exact test or Pearson’s χ^2^ test was used in the categorical variables, and Mann–Whitney U test was used in the nonnormal distribution data to compare the differences between the two groups.

a Only included IVF/ICSI with the husband’s semen.

MMAF, multiple morphological abnormalities of the sperm flagella; ET, embryo transfer; IVF, *in vitro* fertilization; ICSI, intracytoplasmic sperm injection.

Bold values were used to emphasize the statistical significance (P<0.05).

**Figure 3:**
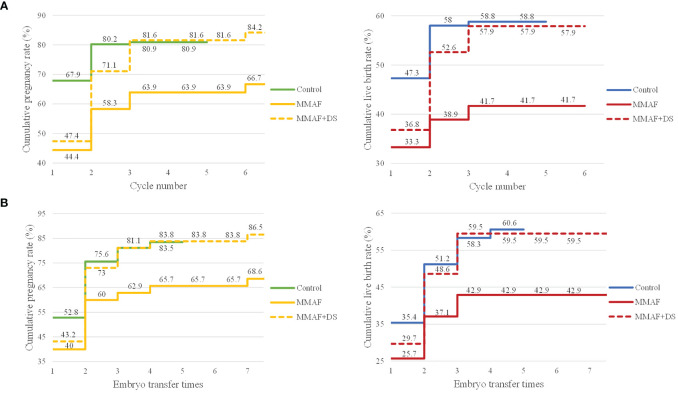
The CLPRs and CLBRs in MMAF, MMAF+donor semen (MMAF+DS), and the control group. **(A)** The CLPR and CLBR for up to six complete cycles in both groups. The difference in CLPRs between the MMAF and control groups was significant (*p* = 0.020). **(B)** The CLPR and CLBR following every embryo transfer time. The log-rank test revealed a significant difference in CLPR between MMAF and the control group (*p* = 0.033). CLPR, cumulative pregnancy rate; CLBR, cumulative live birth rate; MMAF, multiple morphological abnormalities of the sperm flagella; DS, donor semen. Log-rank test and Kaplan–Meier curves with pregnancy and live birth were applied to illustrate differences among groups.

Furthermore, to exhibit in detail the time to pregnancy and successful delivery in the patients with MMAF in ART attempts, CLPR and CLBR following every embryo transfer time and each complete cycle were analyzed and are presented in **Figure 3**. Moreover, to get a generalized reproductive follow-up of these special patients, cycles with donor semen in the MMAF group were also included as MMAF including the donor semen group (MMAF+DS group). In the MMAF group, embryo transfer times and complete cycles were up to seven times and six cycles, respectively. The CLPR following every embryo transfer time rose from 40% to 68.6% in the MMAF group. In the MMAF+DS and control groups, the CLPR was 43.2% and 52.8%, respectively, rising to 86.5% (MMAF+DS) and 83.5% (control) for the last embryo transfer time. The log-rank test revealed a significant difference between the MMAF and control groups (*p* = 0.033). Meanwhile, the CLBR was 25.7% (MMAF), 29.7% (MMAF+DS), and 35.4% (control) after the first embryo transfer time, then increased to 42.9%, 59.5%, and 60.6%, respectively. In the aspect of each complete cycle, the CLPR was 44.4% (MMAF), 47.4% (MMAF+DS), and 67.9% (control) for the first complete cycle, rising correspondingly to 66.7%, 84.2%, and 80.9% for the last cycle. The difference in CLPRs between the MMAF and control groups was significant (*p* = 0.020). The CLBR after the last cycle is 41.7%, 57.9%, and 58.8% in the MMAF, MMAF+DS, and control groups, respectively (*p* = 0.074).

### Neonatal outcomes

All pregnant women were followed up until a live birth was achieved or an abortion occurred. A total of 99 neonatal outcomes from MMAF (*n* = 17, of which 2 were from a twin gestation) and control (*n* = 82, of which 10 were from five twin gestations) are presented in **Table 3**. No significant differences were observed in the weeks of gestation, preterm birth rate, and birth weight between the MMAF and the oligoasthenospermia groups. One baby (5.9%) with a spine defect at live birth was observed among the MMAF cycles, while no malformations were recorded in the oligoasthenospermia group.

**Table 3:** Neonatal outcomes of MMAF and the oligoasthenospermia groups.

	MMAF ^a^ (*n* = 36)	Oligoasthenospermia (*n* = 131)	*p*-value
**No. of newborns**	17	82	–
**Weeks of gestation**	38.6 ± 1.4	38.4 ± 1.6	0.597
**Preterm birth rate**	20.0 (3/15)	14.3 (11/77)	0.864
**Delivery (normal/cesarean section)**	4/11	16/61	0.870
**Gender (female/male)**	8/9	39/43	0.970
**Birth weight (g)**	3,091.8 ± 454.8	3,193.1 ± 566.2	0.490
**No. of birth weight <2,500 *g***			
**Single**	0 (0/13)	4.2 (3/72)	1.000
**Twins**	25.0 (1/4)	40 (4/10)	1.000
**No. of birth defects at live birth**	1	0	–

Continuous variables are presented as the median ± standard deviation. Categorical variables are presented as % (n/N). Fisher’s exact test or Pearson’s χ^2^ test was used in the categorical variables. Mann–Whitney U test was used in the nonnormal distribution data and Student’s t-test was performed for normally distributed data.

a Only included IVF/ICSI with the husband’s semen.

MMAF, multiple morphological abnormalities of the sperm flagella; IVF, *in vitro* fertilization; ICSI, intracytoplasmic sperm injection.

## Discussion

In this study, we analyzed clinical phenotypes, genetic variants, and overall ART outcomes of patients in the cohort of MMAF. The results suggested that nearly half of the patients with MMAF were identified in the pathogenic genetic variants and patients with MMAF presented compromised ART outcomes, including lower embryo developmental potential (decreased fertilization rate, 2PN cleavage rate, blastocyst formation rate, and available blastocyte rate) and impaired clinical outcomes (reduced CLPR), compared with the cohort of oligoasthenozoospermia.

MMAF is a specific kind of asthenoteratozoospermia, which is highly suspected to be associated with genetic mutations (19). WES and Sanger sequencing are common methods to detect abnormal mutations. According to previous studies, WES has uncovered high frequencies of mutations in *DNAH1*, *CFAP44*, and *CFAP43*, which covered approximately one-third of all MMAF cases (5). *AK7*, *CFAP69*, *CEP135*, *AKAP3*, and *AKAP4* were also related to MMAF with strong genetic evidence (5). In the current study, WES was performed in 23 patients with MMAF and presented the mutation in *DNAH1*, *DNAH11*, *CFAP43*, *FSIP2*, and *SPEF2*. A high frequency of mutation in *DNAH1* was in line with the previous conclusion (20). Different gene mutations observed in patients with MMAF might be linked to the common components among the sperm centrosome, the mitotic spindle, and flagella. The SEM showed aberrant flagellar phenotypes with absent, short, bent, coiled, and/or irregular flagella in the MMAF group. According to the TEM, the abnormal ultrastructure of sperm flagella was presented with an absent central complex and/or a disorganized axonemal structure. The identification of genetic mutations combined with ultrastructural evaluation is not only pivotal for revealing mechanisms underlying spermiogenesis in individuals with MMAF but also important for improving treatment choices and providing adequate genetic counseling for patients.

Sperm immobility is the most common feature of individuals with MMAF; thus, affected couples are only able to conceive via ICSI. To date, the relationship between MMAF and ART outcomes has been investigated. Our results with an enlarged sample and strictly statistical comparison showed a significant decrease in fertilization rate, 2PN cleavage rate, blastocyst formation rate, and available blastocyte rate, and presented lower CLPR in the cohort of MMAF. Therefore, the poor ART outcomes exhibited in our study suggest that MMAF may affect the procedure of fertilization and decrease the quality of embryos. A similar association has already been reported between individuals with MMAF carrying *CFAP65*, *DNAH17*, and *CEP135* mutations and compromised ICSI outcomes (21–23). Meanwhile, Fauque et al. (9) observed that infertile patients with ultrastructural abnormalities of the sperm flagellum could disturb early embryonic development. In contrast, some studies presented comparable results in laboratory and clinical outcomes in the cohort of the MMAF and control groups (4, 10, 11, 24). The reason for the inconsistent results may be partly due to the limited sample size and the selection criterion of the control group. Most of the previous studies were case reports or small-scale studies that may be affected by individual differences. In addition, although no significant differences were found in several previous studies, the ART outcomes still declined in the MMAF group. On the other hand, male infertility patients caused by oligoasthenozoospermia were strictly chosen as the control group, as patients with some factors such as testicular sperm extraction (TESE) and total teratozoospermia may affect ICSI outcomes. Moreover, PSM was used to set the control group to eliminate the bias as much as possible, thus resulting in more robust and reliable data.

The reasons for unsatisfactory ART outcomes in the MMAF cohort are worth pondering. Published studies have shown that most of the genetic mutations of MMAF were related to the flagellum (6). The sperm flagellum sustains sperm motility, which is indispensable for sperm progression. Therefore, most patients with MMAF presented immotile sperm, which made it difficult for embryologists to distinguish the viable and dead sperm and then affected the fertilization progress. Currently, chemical substances, such as pentoxifylline and theophylline, which can partly induce sperm motility, were optional effective methods for embryologists to identify and select viable sperm (25, 26). In addition, some causative mutated genes in patients with MMAF, such as *DNAH6* (27), *CFAP65* (12), *SPEF2* (28), and *CFAP69* (6), were reported in connection with abnormal sperm head and acrosome, which is the main Ca^2+^ storage sites in the sperm (29). Ca^2+^ signal is pivotal to sperm function, including hyperactivation, chemotaxis, and acrosome reaction (29, 30). The impairment of acrosome may lead to male subfertility and fertilization failure via Ca^2+^ signal deficiency. Furthermore, MMAF may affect the structure of the sperm, such as chromatin or the integrity of the sperm centrosome, which are essential for regulating syngamy and first mitosis following fertilization and the developmental potential of the embryo (12, 31). Taken together, the compromised ART outcomes in individuals with MMAF were reasonable.

One of the strengths of the current study is the comprehensive assessment of the ART outcomes and a long-term follow-up until the live birth for patients with MMAF, including CLPR, CLBR, the application of donor semen, and time to pregnancy. CLPR and CLBR could provide a more precise estimate of treatment efficacy for couples and summarize the chance of a pregnancy/live birth over an entire ART period, which have been suggested as more suitable ways of evaluating ART outcomes (32, 33). As presented in the study, the CLPRs were significantly lower in the MMAF group and the CLBRs were also below the oligoasthenospermia group, whether in the aspect of embryo transfer times or complete cycles, indicating compromised ART outcomes in the cohort of MMAF. Although patients with MMAF can achieve successful pregnancy via ICSI, more ovarian stimulation cycles are needed to obtain pregnancy according to our results, which means more time and cost will be spent on the ART process. Furthermore, eight cycles with donor semen in patients with MMAF were included in the current study to individually evaluate the ART pregnancy success in these special groups. Because most couples used donor semen from the second complete cycle, the CLPR and CLBR increased substantially from the second complete cycle in the MMAF+DS group, which were similar to the control group and higher than the MMAF group. Remarkably, donor sperm could offer patients with MMAF better ART outcomes. Based on the current results, strong evidence was offered to the clinicians in the counseling and therapy guidance of MMAF. Individualized treatment can be developed depending on the patient’s intentions and demands, including the expectation of successful pregnancy/live birth; concerns about heritage, time, and cost planning on the ART; and the economic condition.

Furthermore, this is the first study to focus on the neonatal outcomes of the MMAF cohort. The present study reported the delivery of healthy babies after transfer with embryos originating from sperm with MMAF. Seventeen babies were born including two pairs of twins in the MMAF group. One case with major malformation was reported in the twin group. In general, the neonatal outcomes of the MMAF group were similar to the control group, which indicated that embryos derived from the MMAF group have the capacity to develop normally and may lead to babies with no major malformations. Studies with larger sample sizes and long-term follow-ups are still needed to determine the effect of MMAF on newborns. Moreover, given that MMAF has a strong relationship with genetic mutations, the potential for transmission of MMAF to offspring is a leading concern. Therefore, patients with MMAF need to be well informed of the possibility of heritable mutations and the risk of infertility.

However, there were still several limitations. Firstly, owing to the low incidence of MMAF, this was a retrospective single-center study with a small sample size. Then, WES was not performed in all patients with MMAF. Further exploration of the potential genetic mutations or underlying mechanisms in patients without identified mutations via WES is needed. The relationship between different gene mutations and the compromised ART outcomes in the MMAF cohort was also worth exploring. Finally, long-term follow-up of the neonates needs to be addressed in the future.

In addition, for patients with MMAF, the prognosis of ICSI treatment may differ among the results of genetic diagnosis; thus, a systematic genetic screening can help physicians not only diagnose with certainty but also provide precise treatment instructions and pregnancy prediction based on the types of genetic mutations. Furthermore, expanding the mutational and phenotypic spectrum of MMAF genes can lay a foundation for the diagnostic screening kits of MMAF.

## Conclusions

In general, genetic mutations account for part of the pathogenesis in MMAF. MMAF may exert deterioration effects on the gamete and embryonic development potential with a compromised ART outcome compared with a male-infertile control group. Results from the analysis of genetics and ART outcomes may provide clinicians with more elaborate and individual evidence in MMAF counseling. Furthermore, large-scale studies and more in-depth mechanism research are still needed to guide future clinical trials.

## Data availability statement

The raw data supporting the conclusions of this article will be made available by the authors, without undue reservation.

## Ethics statement

The studies involving humans were approved by the Ethical Committee of Tongji Hospital, Tongji Medical College, Huazhong University of Science and Technology. The studies were conducted in accordance with the local legislation and institutional requirements. The participants provided their written informed consent to participate in this study.

## Author contributions

RL: Writing – review & editing, Writing – original draft. MW: Writing – review & editing. JZ: Writing – review & editing. RM: Writing – review & editing. CW: Writing – review & editing. LG: Writing – review & editing. YC: Writing – review & editing. LJ: Writing – review & editing. LZ: Writing – review & editing.
