# Supplementary material for: Decreased embryo developmental potential and lower cumulative pregnancy rate in men with multiple morphological abnormalities of the sperm flagella
Source: Front Endocrinol (Lausanne). 2024 Apr 30;15:1377780. doi: 10.3389/fendo.2024.1377780 (PMC11091255; doi:10.3389/fendo.2024.1377780)
Supplement: Supplementary file 1 [file Table_1.docx]

**Supplementary Table 1 Baseline characteristics of MMAF and the oligoasthenospermia groups**

|  | **MMAF (n=36)** | **Oligoasthenospermia (n=131)** | ***P* value** |
| --- | --- | --- | --- |
| **Age (years)** |  |  |  |
| **Female** | 30 (26-32) | 29 (27-32) | 0.675 |
| **Male ^a^** | 31 (28-34) | 31 (29-35) | 0.308 |
| **Female BMI (kg/m^2^)** | 21.7 (19.7-24.9) | 21.6 (20.0-24.9) | 0.712 |
| **Female basal FSH (mIU/mL)** | 7.2 (5.8-8.1) | 6.5 (5.6-7.8) | 0.256 |
| **AFC** | 15 (11-23) | 15 (9-22) | 0.576 |
| **Female AMH (ng/mL)** | 5.3 (2.6-7.0) | 4.6 (2.4-7.6) | 0.757 |
| **Type of infertility** |  |  | 0.572 |
| **Primary** | 36 | 118 |  |
| **Secondary** | 2 | 13 |  |
| **Duration of infertility (years)** | 3 (2-5) | 3 (2-5) | 0.570 |
| **COH protocol** |  |  | 0.238 |
| **GnRH-agonist** | 28 | 82 |  |
| **GnRH-antagonist** | 23 | 69 |  |
| **Others** | 2 | 20 |  |
| **Gn duration (days)** | 10 (9-11) | 9 (8-11) | 0.230 |
| **Gn dosage (IU)** | 2025.0 (1650.0-2700.0) | 2125.0 (1500.0-2721.8) | 0.882 |
| **No. of large follicles on hCG day** | 12 (9-16) | 11 (6-15) | 0.134 |
| **Estradiol level on hCG day (pg/mL)** | 3000.0 (2078.0-3205.0) | 2282.5 (1473.5-3525.5) | 0.065 |
| **Progesterone level on hCG day (pg/mL)** | 1.0 (0.8-1.1) | 0.9 (0.5-1.1) | 0.101 |
| **Endometrium thickness on hCG day (mm)** | 11.4 (10.4-13.1) | 11.2 (9.5-12.9) | 0.172 |
| **Semen characteristics ^a^** |  |  |  |
| **Sperm concentration (10^6^/mL)** | 11.4 (7.4-25.3) | 11.6 (8.4-14.0) | - |
| **Total motility (%**) | 0.2 (0-10.6) | 25.5 (17.1-37.0) | - |
| **Progressive motility (%)** | 0 (0-8.1) | 21.9 (13.7-26.3) | - |
| **Morphologically normal forms (%)** | 1.2 (0.6-3.1) | 5 (4.2-6.0) | - |

Note: Continuous variables are presented as the median (interquartile range). Mann–Whitney U test was used in the nonnormal distribution data and Student’s t-test was performed for normally distributed data.

MMAF: multiple morphological abnormalities of the sperm flagella, BMI: body mass index, FSH: follicle-stimulating hormone, AFC: antral follicle count, AMH: antimüllerian hormone, hCG: human chorionic gonadotropin, COH: controlled ovarian hyperstimulation, GnRH: gonadotropin-releasing hormone, Gn: gonadotropin.

^a^ Including two patients only use donor semen throughout the whole IVF/ICSI procedure.
