# Supplementary material for: Decreased embryo developmental potential and lower cumulative pregnancy rate in men with multiple morphological abnormalities of the sperm flagella
Source: Front Endocrinol (Lausanne). 2024 Apr 30;15:1377780. doi: 10.3389/fendo.2024.1377780 (PMC11091255; doi:10.3389/fendo.2024.1377780)
Supplement: Supplementary file 2 [file Table_2.docx]

**Supplement Table 2 Mutations of genes in the MMAF group**

| Patient | Gene | Mutation | Zygosity | Type | AD/AR | Allele frequency ^a^ | Clinical significance ^b^ | SIFT | Polyphen-2 | MutationTaster | SpliceAI |
| --- | --- | --- | --- | --- | --- | --- | --- | --- | --- | --- | --- |
| e | *DNAH1*  NM_015512 | c.10627-3C>G [NA]  c.11957A>T [p.E3986V] | Heterozygous | Splicing  Missense | AR | 0.000004037  - | Uncertain significance  Uncertain significance | -  Damaging | -  Probably damaging | -  Disease causing | 0.68  - |
| P2 |  | c.10627-3C>G [NA]  c.11726_11727delCT [p.P3909fs] | Heterozygous | Splicing  Frameshift | AR | 0.000004037  0.0001007 | Uncertain significance  Pathogenic | -  - | -  - | -  - | 0.68  - |
| P3 |  | c.7428delC [p.R2477fs]  c.11726_11727delCT [p.P3909fs] | Heterozygous | Frameshift  Frameshift | AR | -  0.0001007 | Pathogenic  Pathogenic | -  - | -  - | -  - | -  - |
| P4 |  | c.7435C>T [p.R2479X]  c.10757T>C [p.F3586S] | Heterozygous | Missense  Missense | AR | 0.00001292  0.00002032 | Likely pathogenic  Uncertain significance | -  Damaging | -  Probably damaging | Disease causing automatic  Disease causing | -  - |
| P5 |  | c.11726_11727delCT [p.P3909fs]  c.12154delC [p.L4052fs] | Heterozygous | Frameshift  Frameshift | AR | 0.0001007  - | Pathogenic  Likely pathogenic | -  - | -  - | -  - | -  - |
| P6 |  | c.4875-19A>G [NA] | Homozygous | Splicing | AR | 0.000004825 | Uncertain significance | - | - | - | 0.99 |
| P7 |  | c.799G>T [p.E267X]  c.2016T>G [p.Y672X] | Heterozygous | Missense  Missense | AR | -  - | Likely pathogenic  Likely pathogenic | -  - | -  - | Disease causing automatic  Disease causing automatic | -  - |
| P8 | *DNAH11*  NM_001277115 | c.9017C>T [p.T3006M]  c.11255A>C [p.D3752A] | Heterozygous | Missense  Missense | AR | 0.00008202  0.00002818 | Uncertain significance  Uncertain significance | Damaging  Damaging | Probably damaging  Probably damaging | Disease causing  Disease causing | -  - |
| P9 | *CFAP43*  NM_025145 | c.4132C>T [p.R1378X]  c.2802T>A [p.C934X] | Heterozygous | Missense  Missense | AR | -  - | Likely pathogenic  Likely pathogenic | -  - | -  - | Disease causing automatic  Disease causing automatic | -  - |
| P10 | *FSIP2*  NM_173651 | c.1494C>A [p.C498X]  c.11280_11284delTAAGT [p.I3761fs] | Heterozygous | Missense  Frameshift | AR | -  0.00007157 | Pathogenic  Pathogenic | -  - | -  - | Disease causing automatic  - | -  - |
| P11 | *SPEF2*  NM_024867 | c.3400delA [p.I134fs] | Homozygous | Frameshift | AR | 0.000004011 | Likely pathogenic | - | - | - | - |

Note: ^a^ the allele frequency were analyzed by gnomAD.

^b^ the clinical significance was analyzed via American College of Medical Genetics and Genomics (ACMG).
